# Supplementary material for: The positive effect of plant diversity on soil carbon depends on climate
Source: Nat Commun. 2023 Oct 19;14:6624. doi: 10.1038/s41467-023-42340-0 (PMC10587103; doi:10.1038/s41467-023-42340-0)
Supplement: Supplementary file 1 — Supplementary Information [file 41467_2023_42340_MOESM1_ESM.pdf]

Supplementary Information

The positive effect of plant diversity on soil carbon depends on climate

Marie Spohn[1*], Sumanta Bagchi[2], Lori A. Biederman[3], Elizabeth T. Borer[4], Kari Anne Bråthen[5], Miguel N. Bugalho[6], Maria C. Caldeira[7] , Jane A. Catford[8, 9], Scott L. Collins[10], Nico Eisenhauer[11, 12], Nicole Hagenah[13], Sylvia Haider[11,14,15], Yann Hautier[16], Johannes M. H. Knops[17], Sally E. Koerner[18], Lauri Laanisto[19], Ylva Lekberg[20], Jason P. Martina[21], Holly Martinson[22], Rebecca L. McCulley[23], Pablo L. Peri[24], Petr Macek[25], Sally A. Power[26], Anita C. Risch[27], Christiane Roscher[28,11], Eric W. Seabloom[4], Carly Stevens[29], G.F. (Ciska) Veen[30], Risto Virtanen[31], Laura Yahdjian[32]

[1]Dept of Soil and Environment, Swedish University of Agricultural Sciences (SLU), Lennart Hjelms väg 9, 75007 Uppsala, Sweden
[2]Indian Institute of Science, Bangalore 560012, India
[3]Dept of Ecology, Evolution, and Organismal Biology, Iowa State University, 251 Bessey Hall, Ames IA 50011, USA
[4]Dept of Ecology, Evolution, and Behavior, University of Minnesota, St Paul, MN, USA
[5]Dept of Arctic and Marine Biology, UiT – Arctic University of Norway, Tromsø, Norway
[6]Centre for Applied Ecology "Prof. Baeta Neves" (CEABN-InBIO), School of Agriculture, University of Lisbon, Portugal
[7]Forest Research Centre, Associate Laboratory TERRA, School of Agriculture, University of Lisbon, Portugal
[8]Dept of Geography, King's College London, 30 Aldwych, London, WC2B 4BG, UK
[9]School of Agriculture, Food and Ecosystem Sciences, University of Melbourne, Parkville, Vic 3010, Australia
[10]Dept of Biology, University of New Mexico, Albuquerque, NM 87131, USA
[11]German Centre for Integrative Biodiversity Research (iDiv) Halle-Jena-Leipzig, Puschstraße 4, 04103 Leipzig, Germany
[12]Leipzig University, Institute of Biology, Puschstraße 4, 04103 Leipzig, Germany
[13]Mammal Research Institute, Dept of Zoology & Entomology, University of Pretoria, Pretoria, South Africa
[14]Leuphana University of Lüneburg, Institute of Ecology, Universitätsallee 1, 21335 Lüneburg, Germany
[15]Martin Luther University Halle-Wittenberg, Institute of Biology and Geobotany and Botanical Garden, Am Kirchtor 1, 06108 Halle, Germany
[16]Ecology and Biodiversity Group, Dept of Biology, Utrecht University, Padualaan 8, 3584 CH Utrecht, The Netherlands
[17]Health and Environmental Sciences, Xián Jiaotong-Liverpool University, Suzhou, China
[18]Dept of Biology, University of North Carolina Greensboro, Greensboro, NC, USA
[19]Dept of Biodiversity and Nature Tourism, Estonian University of Life Sciences, Kreutzwaldi St. 5, 51006, Tartu, Estonia
[20]MPG Ranch and University of Montana, Montana, USA
[21]Dept of Biology, Texas State University, San Marcos, TX 78666, USA
[22]Dept of Biology, McDaniel College, Westminster, MD 21157, USA
[23]Dept of Plant & Soil Sciences, University of Kentucky, Lexington, KY, 40546, USA
[24]National Institute of Agricultural Technology (INTA), Rio Gallegos, Santa Cruz, Argentina

[25]Institute of Hydrobiology, Biology Centre of the Czech Academy of Sciences, Na Sadkach 7, 370 05 Ceske Budejovice, Czech Republic
[26]Haweksbury Institute for the Environment, Locked Bag 1797, Penrith, NSW, 2751, Australia
[27]Swiss Federal Institute for Forest, Snow and Landscape Research WSL, Zuercherstrasse 111, 8903 Birmensdorf, Switzerland
[28]UFZ, Helmholtz Centre for Environmental Research, Dept Physiological Diversity, Permoserstrasse 15, 04318 Leipzig, Germany
[29]Lancaster Environment Centre, Lancaster University, Lancaster, LA1 4YQ, UK
[30]Dept of Terrestrial Ecology, Netherlands Institute of Ecology, Droevendaalsesteeg 10, 6708 PB, Wageningen, the Netherlands
[31]Ecology & Genetics, University of Oulu, PO Box 3000, 90014 University of Oulu, Finland
[32]Instituto de Investigaciones Fisiológicas y Ecológicas Vinculadas a la Agricultura (IFEVA), CONICET, Faculty of Agronomy, University of Buenos Aires, Argentina

*Corresponding author. Email: marie.spohn@slu.se

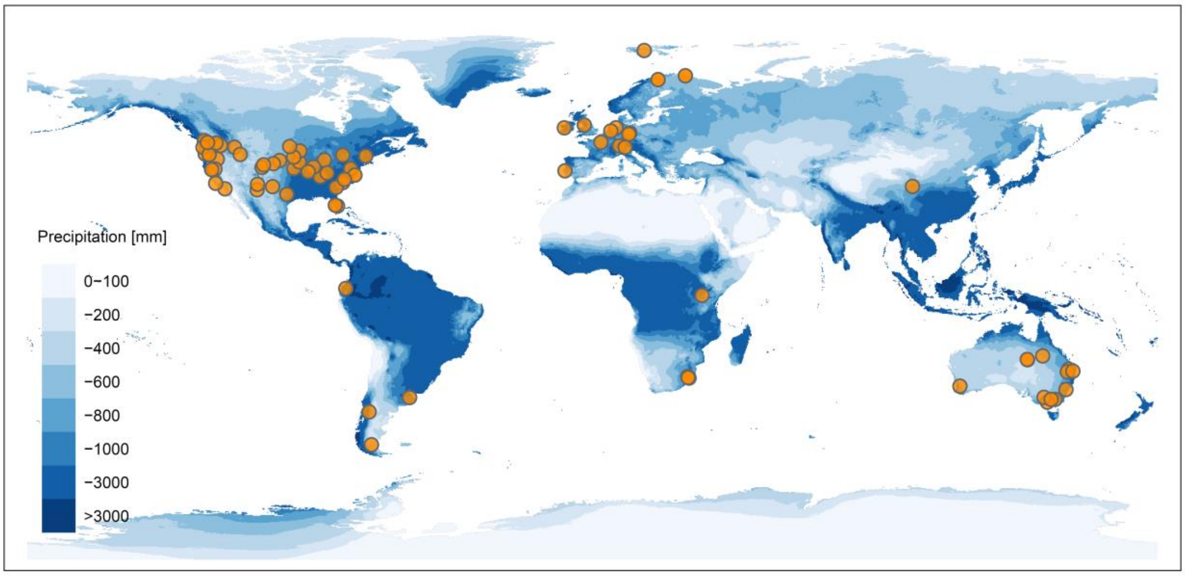

Fig. S1. Global map depicting mean annual precipitation and the 84 grassland sites (orange dots). The map was created in R (version 4.2.1) using the packages ggmap (version 3.0.2) and OpenStreetMap (version 0.3.4).

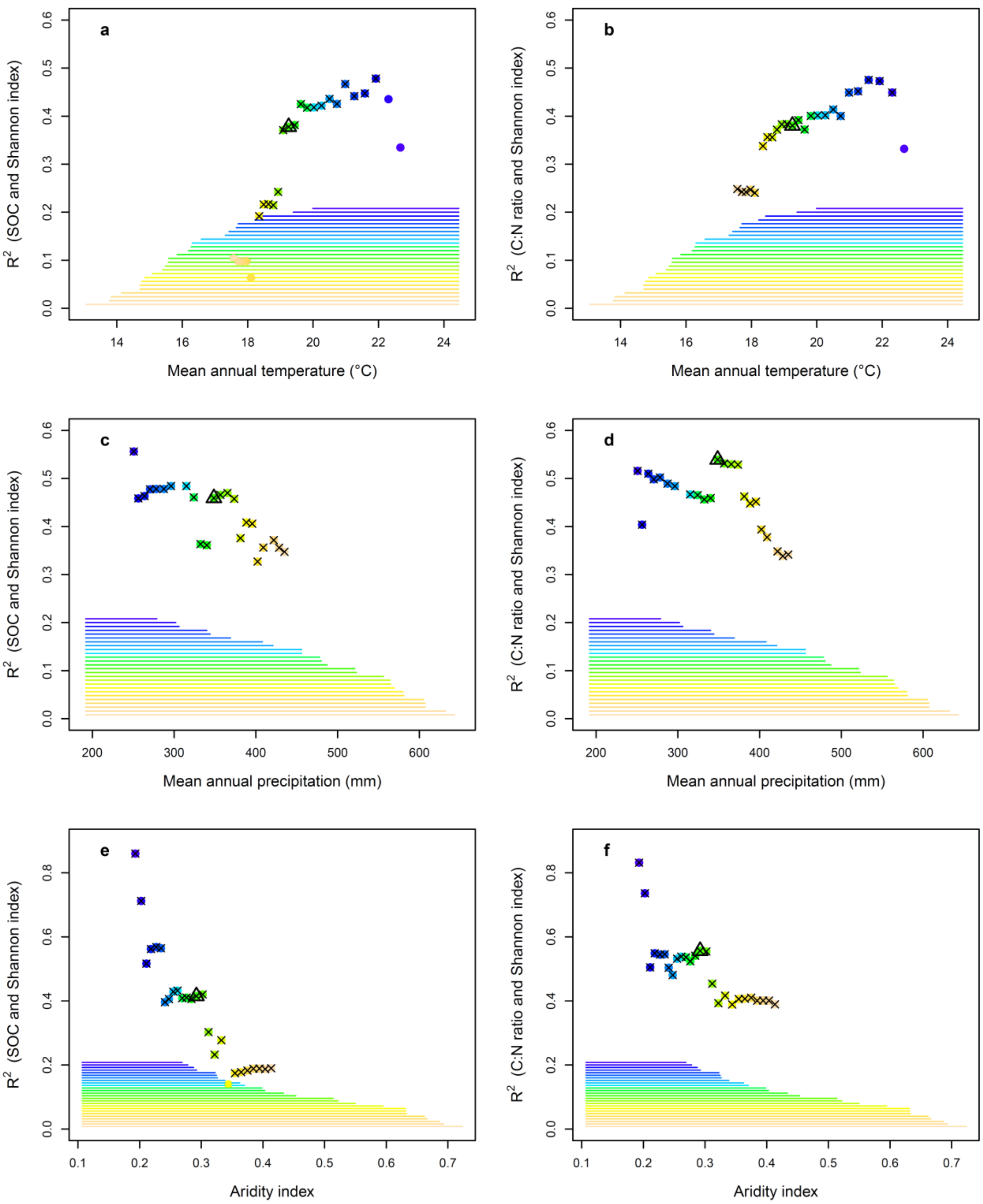
a
b
c
d
e
f
R2 (SOC and Shannon index)
R2 (C:N ratio and Shannon index)
Mean annual temperature (°C)
Mean annual precipitation (mm)
Aridity index

Fig. S2. Coefficient of determination (R^2) of linear models of soil organic carbon (SOC) and Shannon index as well as carbon-to-nitrogen (C:N) ratio and Shannon index for different subsets of sites selected according to climate variables. *Points* depict the R^2 of the linear model of SOC and Shannon index (a, c, e) as well as C:N ratio and Shannon index (b, d, f) for 26 subsets of sites as a function of the mean of the climate variable of each subset (mean annual temperature (MAT; a, b), mean annual precipitation (MAP; c, d), and aridity index (e, f)). The smallest subset in each panel (marked in blue) consists of eight sites with either the highest MAT, lowest MAP or lowest aridity index. The size of the subsets was increased in a stepwise manner by decreasing MAT or by increasing MAP or aridity index (adding one site per step). The largest subset (marked in beige) consists of 33 sites. Points marked by a cross (×) indicate statistically significant models ($P < 0.05$). The point of each panel that is marked by a black triangle corresponds to the linear model shown in Figure 2 or 3. *Horizontal lines* at the bottom of the panels show the MAT, MAP or aridity index ranges of each of the 26 subsets. The horizontal line and the point of each subset have the same color. Note that by definition, the aridity index increases with decreasing aridity.

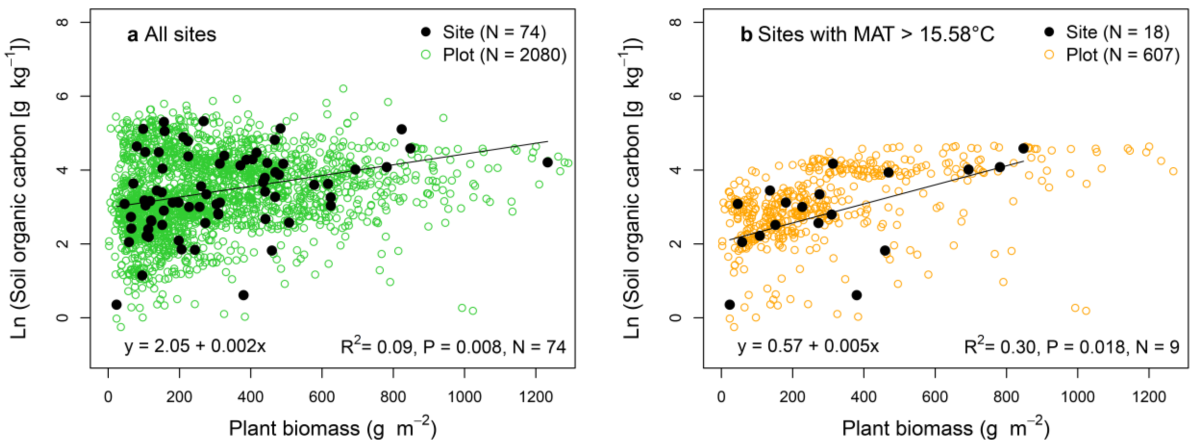

Fig. S3. Relationship between plant biomass and soil organic carbon content. The relationship is shown across all grassland sites (a) as well as across sites with mean annual temperature (MAT) > 15.58 °C (b). The linear models were plotted to the site-level data (and not to the plot data, which is shown to give insight into the variability).

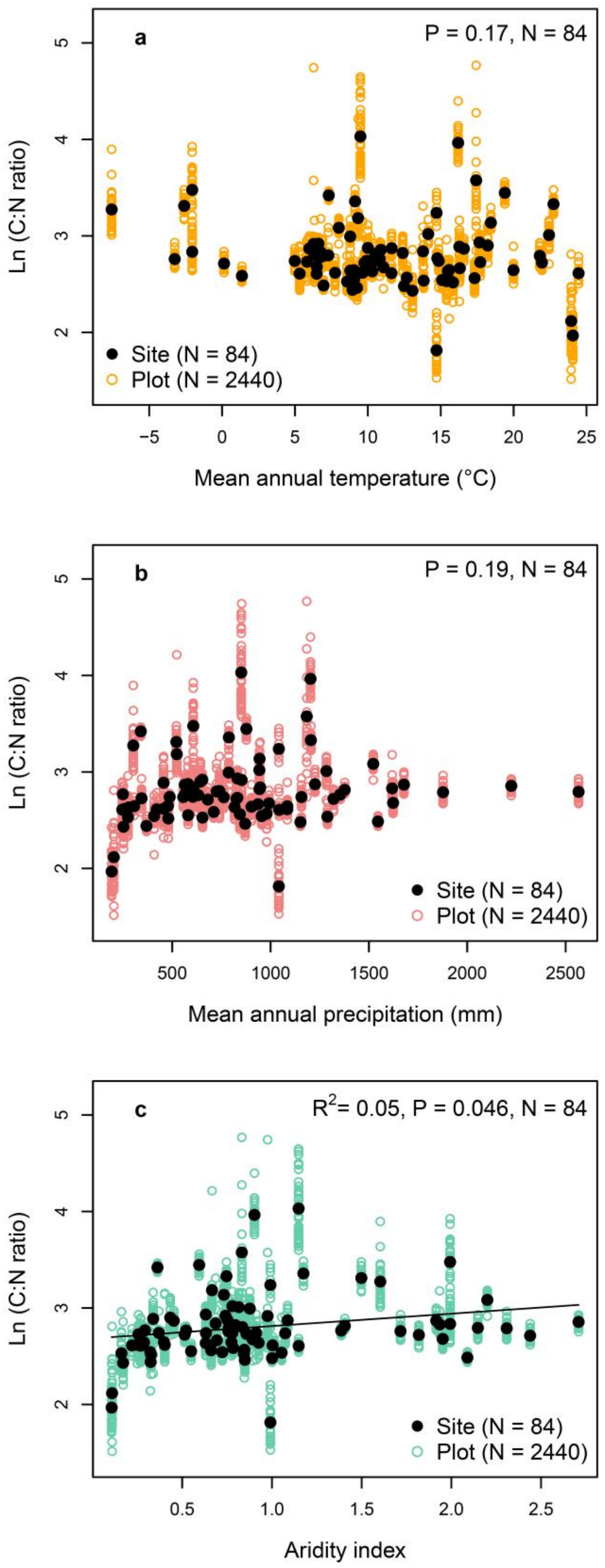

Fig. S4. Soil total organic carbon-to-nitrogen (C:N) ratio as a function of climate.
Soil C: N ratio as a function of mean annual temperature (a), mean annual precipitation (b), and the aridity index (c) across 84 grasslands. The linear mode was plotted to the site-level data (and not to the plot data, which is shown to give insight into the variability).

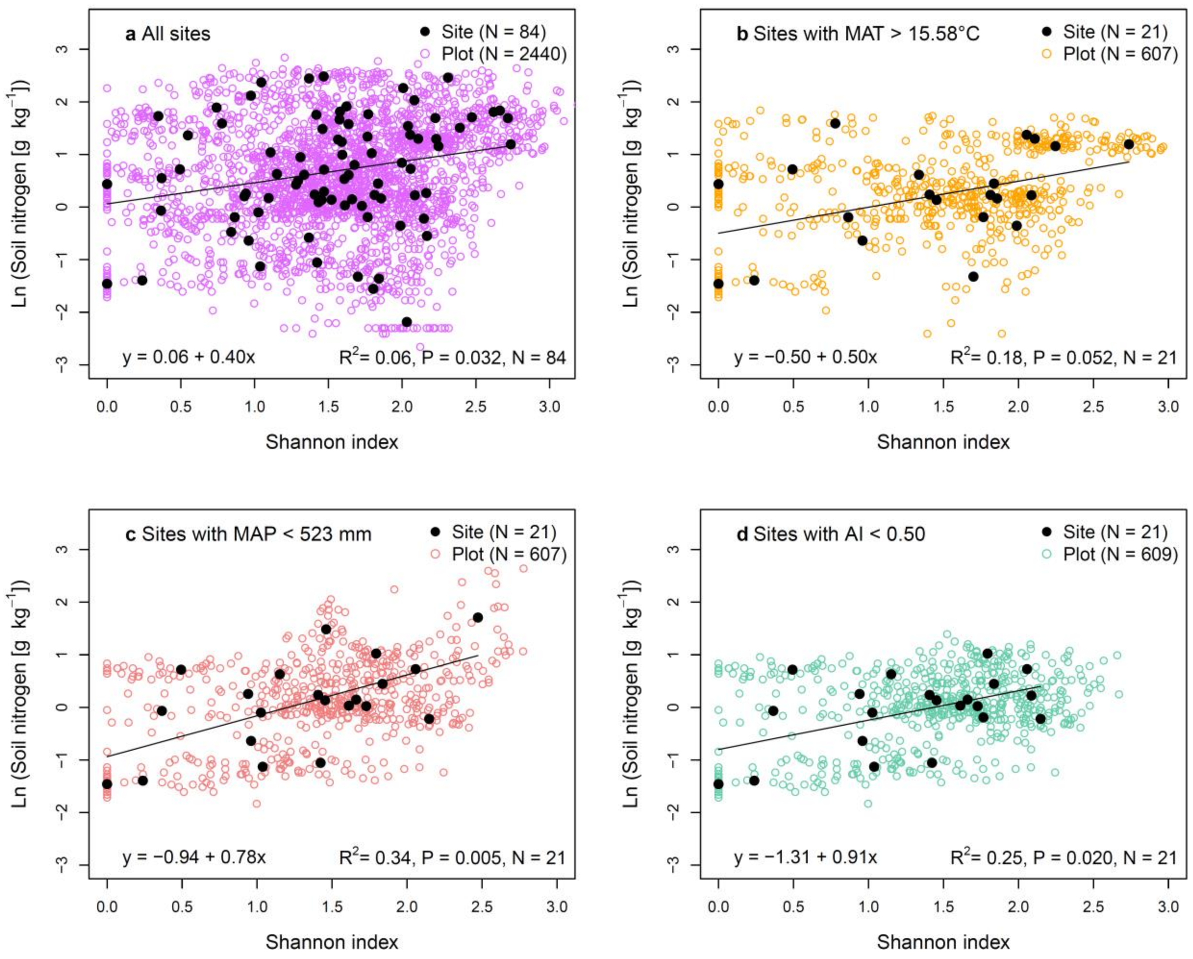

Fig. S5. Relationship between the Shannon index and soil nitrogen content. The relationship is shown across all 84 grassland sites (a) as well as across sites with mean annual temperature (MAT) > 15.58 °C (b), sites with mean annual precipitation (MAP) < 523 mm (c), and arid and semi-arid sites, i.e., sites with an aridity index (AI) < 0.50 (d). The linear models were plotted to the site-level data (and not to the plot data, which is shown to give insight into the variability).

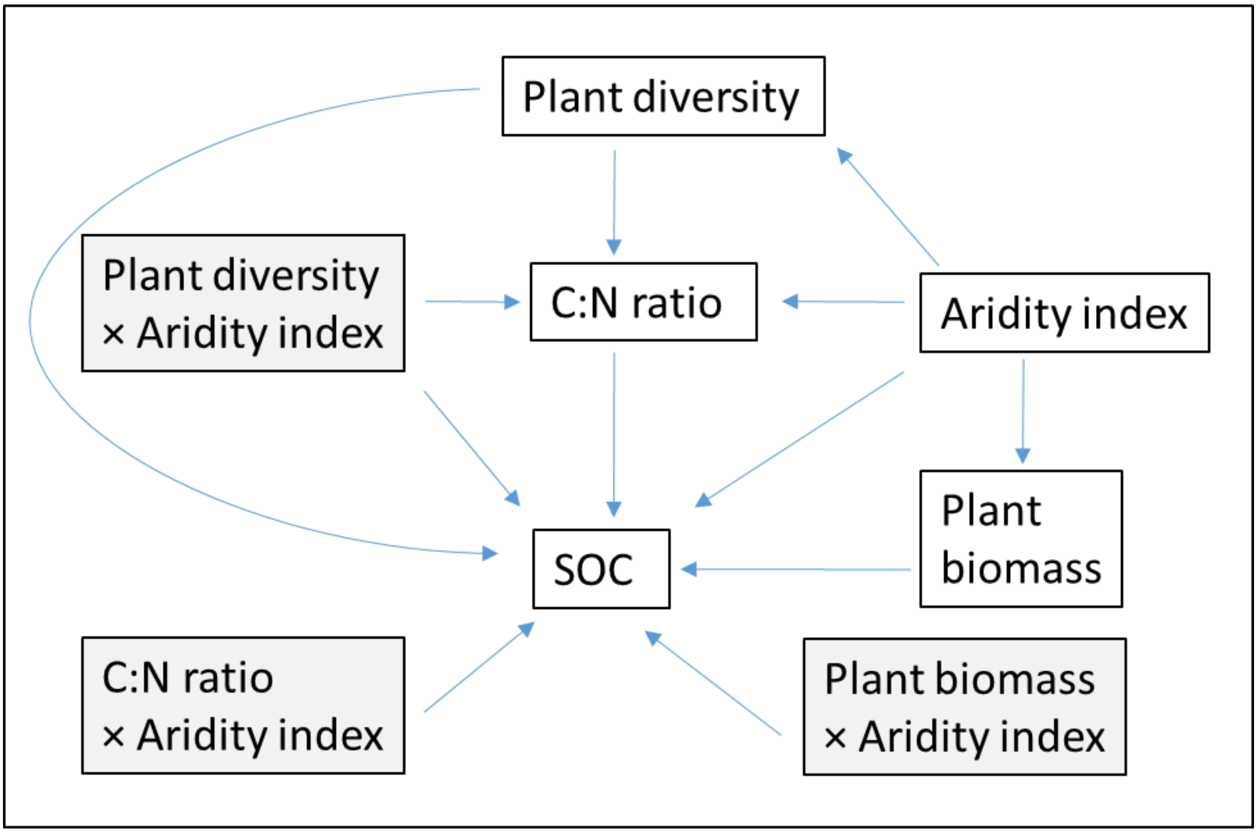

Fig. S6. Initial version of the new structural equation model.

Table S1. All 84 grassland sites ordered by continent, together with their mean annual temperature (MAT), mean annual precipitation (MAP), aridity index, and number of plots.

Continent	Site name (including country code)	Grassland type	MAT (°C)	MAP (mm)	Aridity index	Number of plots
Africa	gilb.za	montane grassland	14.14	943	0.78	11
Africa	sereng.tz	savanna	21.94	827	0.51	28
Africa	summ.za	mesic grassland	18.44	944	0.73	30
Africa	ukul.za	mesic grassland	17.65	832	0.63	30
Asia	azi.cn	alpine grassland	1.36	711	0.78	29
Australia	bogong.au	alpine grassland	5.98	1678	1.92	30
Australia	bunya.au	grassland	15.49	817	0.63	30
Australia	burrawan.au	semiarid grassland	18.22	643	0.43	30
Australia	derr.au	semiarid grassland	14.77	565	0.52	13
Australia	ethamc.au	desert grassland	24.06	192	0.11	26
Australia	ethass.au	desert grassland	23.95	203	0.11	30
Australia	kiny.au	semiarid grassland	15.59	408	0.32	30
Australia	mitch.au	semiarid grassland	24.45	421	0.22	10
Australia	nilla.au	old field	13.78	947	0.69	30
Australia	ping.au	old field	16.28	456	0.34	30
Australia	pinj.au	pasture	19.99	1085	0.77	30
Australia	yarra.au	mesic grassland	17.32	844	0.66	40
Europe	badlau.de	old field	9.33	523	0.67	24
Europe	burren.ie	calcareous grassland	9.77	1320	1.82	30
Europe	cereep.fr	old field	10.82	632	0.76	30
Europe	comp.pt	annual grassland	16.58	564	0.45	31
Europe	frue.ch	pasture	6.96	1546	2.09	30
Europe	jena.de	grassland	8.57	654	0.84	24
Europe	kilp.fi	tundra grassland	-3.25	569	1.72	38
Europe	lancaster.uk	mesic grassland	8.01	1522	2.20	26
Europe	pape.de	old field	9.12	788	1.18	10
Europe	saana.fi	montane grassland	-2.60	521	1.50	30
Europe	sval.no	tundra grassland	-7.57	302	1.60	29
Europe	valm.ch	alpine grassland	0.13	681	2.44	27
Europe	vargrass.no	tundra grassland	-2.05	607	1.99	23
Europe	varheath.no	heathland	-2.05	607	1.99	24
Europe	veluwe.nl	old field	9.49	851	1.15	50
North America	amcamp.us	mesic grassland	10.00	580	0.75	30
North America	arch.us	mixedgrass prairie	22.73	1205	0.75	30
North America	barta.us	mixedgrass prairie	9.07	581	0.55	30
North America	bldr.us	shortgrass prairie	9.90	487	0.37	20
North America	bnbt.us	tallgrass prairie	12.39	944	0.80	30
North America	bnch.us	montane grassland	6.77	1618	1.93	30
North America	bttr.us	montane grassland	6.50	1623	1.95	30
North America	cammead.us	grassland	6.61	653	0.74	10

North America	cbgb.us	tallgrass prairie	9.26	871	0.85	54
North America	cdcr.us	tallgrass prairie	6.34	740	0.84	47
North America	cdpt.us	shortgrass prairie	9.64	456	0.40	60
North America	cowi.ca	old field	10.45	762	1.07	30
North America	elliot.us	annual grassland	17.71	344	0.26	30
North America	fnly.us	mesic grassland	11.63	1226	1.09	17
North America	glac.us	mesic grassland	10.55	1354	1.39	30
North America	glacphr.us	grassland	10.53	1377	1.41	10
North America	hall.us	tallgrass prairie	13.83	1289	1.05	30
North America	hart.us	shrub steppe	7.75	259	0.25	30
North America	hnvr.us	old field	6.49	1044	1.15	30
North America	hogtwo.us	coastal grassland	14.71	1042	0.99	24
North America	jorn.us	semiarid grassland	15.40	275	0.16	30
North America	kbs.us	old field	8.80	903	0.93	50
North America	koffler.ca	pasture	6.28	853	0.98	36
North America	lake.us	tallgrass prairie	7.29	726	0.76	30
North America	lead.us	salt marsh	10.90	2224	2.71	30
North America	look.us	montane grassland	6.90	1877	2.31	30
North America	lubb.us	semiarid grassland	15.85	480	0.33	42
North America	marcel.us	grassland	9.05	306	0.29	10
North America	mcdan.us	grassland	11.62	1085	1.00	36
North America	meto.us	coastal grassland	14.71	1042	0.99	24
North America	mrapids.us	grassland	10.30	279	0.28	10
North America	msla.us	grassland	7.32	340	0.36	30
North America	msum.us	tallgrass prairie	4.99	556	0.63	30
North America	sage.us	montane grassland	5.83	831	0.86	30
North America	sandhill.us	svanna	16.19	1203	0.90	30
North America	sava.us	svanna	17.43	1184	0.83	20
North America	sedg.us	annual grassland	15.58	478	0.40	30
North America	sevi.us	desert grassland	13.06	252	0.17	40
North America	sgs.us	shortgrass prairie	8.95	369	0.32	16
North America	shps.us	shrub stepp	5.32	246	0.27	38
North America	sier.us	annual grassland	16.31	936	0.69	30
North America	smith.us	mesic grassland	10.18	605	0.78	30
North America	spin.us	pasture	12.48	1152	1.00	30
North America	temple.us	tallgrass prairie	19.40	877	0.59	26
North America	trel.us	tallgrass prairie	11.07	992	0.89	30
North America	tyso.us	old field	12.68	980	0.85	40
North America	ufrec.us	grassland	22.43	1284	0.82	24
North America	unc.us	old field	14.86	1157	0.91	30
South America	bari.ar	grassland steppe	8.79	787	0.88	30
South America	chilcas.ar	mesic grassland	15.09	955	0.72	27
South America	pich.ec	alpine grassland	21.80	2566	2.15	30
South America	potrok.ar	semiarid grassland	6.62	249	0.29	36

Table S2. Results of multiple regression analyses of soil organic carbon as a function of biomass and climate variables. Significant interactions (P < 0.05) between biomass and climate variables are marked in bold font. SOC stands for soil organic carbon, MAT stands for mean annual temperature, MAP stands for mean annual precipitation, AI stands for aridity index. N refers to the number of observations (i.e. number of grassland sites).

Regression	P value	Adjusted R^2	P value Biomass	P value climate variable	P value interaction: Biomass × climate variable	N
Ln(SOC) ~ Biomass × MAT	< 0.001	0.31	0.876	< 0.001	0.078	74
Ln(SOC) ~ Biomass × MAP	< 0.001	0.30	0.003	<0.001	**0.014**	74
Ln(SOC) ~ Biomass × AI	< 0.001	0.49	0.002	< 0.001	**0.010**	74

Table S3. Author contributions according to the guidelines of the Nutrient Network

Name	Developed and framed research question(s)	Analyzed data	Contributed to data analyses	Wrote the paper	Contributed to paper writing	Site level coordinator	Nutrient Network Coordinators
Marie Spohn	×	×		×			
Sumanta Bagchi					×	×	
Lori A. Biederman					×	×	
Elizabeth T. Borer					×	×	×
Kari Anne Bråthen					×	×	
Miguel N. Bugalho					×	×	
Maria C. Caldeira					×	×	
Jane A. Catford					×	×	
Scott Collins					×	×	
Nico Eisenhauer					×	×	
Nicole Hagenah					×	×	
Sylvia Haider					×	×	
Yann Hautier					×	×	
Johannes M. H. Knops					×	×	
Sally E. Koerner					×	×	
Lauri Laanisto					×	×	
Ylva Lekberg					×	×	
Jason P. Martina					×	×	
Holly Martinson					×	×	
Rebecca L. McCulley					×	×	
Pablo L. Peri					×	×	
Macek Petr					×	×	
Sally A. Power					×	×	
Anita C. Risch					×	×	
Christiane Roscher					×	×	
Eric W. Seabloom					×	×	×
Carly Stevens					×	×	
G.F. (Ciska) Veen					×	×	
Risto Virtanen					×	×	
Laura Yahdjian					×	×	
