# Supplementary material for: The positive effect of plant diversity on soil carbon depends on climate
Source: Nat Commun. 2023 Oct 19;14:6624. doi: 10.1038/s41467-023-42340-0 (PMC10587103; doi:10.1038/s41467-023-42340-0)
Supplement: Supplementary file 3 — Reporting Summary [file 41467_2023_42340_MOESM3_ESM.pdf]

nature portfolio

Corresponding author(s): Marie Spohn

Last updated by author(s): Sep 20, 2023

Reporting Summary

Nature Portfolio wishes to improve the reproducibility of the work that we publish. This form provides structure for consistency and transparency in reporting. For further information on Nature Portfolio policies, see our Editorial Policies and the Editorial Policy Checklist.

Statistics

For all statistical analyses, confirm that the following items are present in the figure legend, table legend, main text, or Methods section.

n/a	Confirmed	
☐	☒	The exact sample size (*n*) for each experimental group/condition, given as a discrete number and unit of measurement
☐	☒	A statement on whether measurements were taken from distinct samples or whether the same sample was measured repeatedly
☐	☒	The statistical test(s) used AND whether they are one- or two-sided<br>*Only common tests should be described solely by name; describe more complex techniques in the Methods section.*
☐	☒	A description of all covariates tested
☐	☒	A description of any assumptions or corrections, such as tests of normality and adjustment for multiple comparisons
☐	☒	A full description of the statistical parameters including central tendency (e.g. means) or other basic estimates (e.g. regression coefficient) AND variation (e.g. standard deviation) or associated estimates of uncertainty (e.g. confidence intervals)
☐	☒	For null hypothesis testing, the test statistic (e.g. *F*, *t*, *r*) with confidence intervals, effect sizes, degrees of freedom and *P* value noted<br>*Give P values as exact values whenever suitable.*
☒	☐	For Bayesian analysis, information on the choice of priors and Markov chain Monte Carlo settings
☒	☐	For hierarchical and complex designs, identification of the appropriate level for tests and full reporting of outcomes
☒	☐	Estimates of effect sizes (e.g. Cohen's *d*, Pearson's *r*), indicating how they were calculated

Our web collection on statistics for biologists contains articles on many of the points above.

Software and code

Policy information about availability of computer code

Data collection	No code was used for data collection.
Data analysis	All data analyses were conducted using R (version 4.2.1).<br>We used the R packages VEGAN (version 2.6-4) and piecewiseSEM (version 2.3.0).<br><br>All R code for all analyses conducted in this study is available at this repository.<br>https://doi.org/10.5281/zenodo.8308135

For manuscripts utilizing custom algorithms or software that are central to the research but not yet described in published literature, software must be made available to editors and reviewers. We strongly encourage code deposition in a community repository (e.g. GitHub). See the Nature Portfolio guidelines for submitting code & software for further information.

Data

Policy information about availability of data

All manuscripts must include a data availability statement. This statement should provide the following information, where applicable:

- Accession codes, unique identifiers, or web links for publicly available datasets
- A description of any restrictions on data availability
- For clinical datasets or third party data, please ensure that the statement adheres to our policy

All data are available at this repository.
https://doi.org/10.5281/zenodo.8308135

Research involving human participants, their data, or biological material

Policy information about studies with human participants or human data. See also policy information about sex, gender (identity/presentation), and sexual orientation and race, ethnicity and racism.

Reporting on sex and gender	N/A
Reporting on race, ethnicity, or other socially relevant groupings	N/A
Population characteristics	N/A
Recruitment	N/A
Ethics oversight	N/A

Note that full information on the approval of the study protocol must also be provided in the manuscript.

Field-specific reporting

Please select the one below that is the best fit for your research. If you are not sure, read the appropriate sections before making your selection.

☐ Life sciences ☐ Behavioural & social sciences ☒ Ecological, evolutionary & environmental sciences

For a reference copy of the document with all sections, see nature.com/documents/nr-reporting-summary-flat.pdf

Ecological, evolutionary & environmental sciences study design

All studies must disclose on these points even when the disclosure is negative.

Study description	In this study, we explored the relationship between soil organic carbon content, plant diversity, plant biomass, and climate.<br>The 84 grassland sites analyzed in this study are natural and semi-natural grasslands located on six continents, covering a wide range of climatic conditions (Table S1, Fig. S1). Across the grassland sites, MAT ranges from -7.57 °C to 24.45 °C, MAP ranges from 192 mm to 2566 mm, and the aridity index ranges from 0.107 to 2.709. The 84 sites represent 19 grassland types (Table S1, Fig. S1). All 84 sites are part of the Nutrient Network Global Research Cooperative (1) (NutNet, https://nutnet. org; Borer et al., 2014). For this study, we choose data that were collected in the year before any experimental treatment started, which means that the sites were not experimentally manipulated at the time of data collection.<br>At each of the 84 sites, on average 30 plots, (see Table S1) were analyzed (see Data collection) to gain representative data for each site.
Research sample	The dataset covers 84 grassland sites on six continents, and it includes 19 types of grassland (Table S1). At each of the 84 sites, on average 30 plots (see Table S1) were analyzed (see Data collection) to gain representative data for each site.
Sampling strategy	For this study, we analyzed 84 grassland sites representing 19 types of grassland, located on all continents (except Antarctica). At each site, an average of 30 plots (measuring 5 × 5 m) were examined to gain representative data for each site. No specific sample size calculation was performed.
Data collection	Data collection was led by the authors of this study together with technical staff.<br>Plant species diversity (called plant diversity hereafter) was determined in a randomly designated 1×1m subplot within each 5×5m plot at peak biomass. In the 1×1m subplot, cover was estimated visually to the nearest 1% for every species overhanging the subplot. Data on plant diversity were collected at all 84 sites.<br>Live vascular plant aboveground biomass (called plant biomass hereafter) was estimated destructively by clipping at ground level all aboveground biomass of plants rooted within two 1×0.1m strips (for a total of 0.2m2) adjacent to the 1×1m subplot where plant species diversity was determined. All biomass was dried at 60°C to constant mass before weighing to the nearest 0.01g. Data on plant biomass were collected at 74 of the 84 sites.

Soil samples were collected in the 5 × 5 m plots by taking three soil cores (2.5 cm diameter) at a depth of 0–10 cm. The three cores were pooled to make one sample per plot, air-dried and analyzed for total organic carbon (called soil organic carbon or soil carbon hereafter) and total nitrogen using an elemental analyzer (Costech ECS 4010 CHNSO Analyzer). Soil texture, expressed as the percentage sand, percentage silt, and percentage clay, was measured on 100 g dry soil using the Bouyoucos method. All soil samples were analyzed in the same laboratory (A&L Analytical Laboratory). Data on soil organic carbon (SOC) and soil nitrogen were collected at all 84 sites, data on soil texture were collected at 62 sites.
Plant species diversity (called plant diversity hereafter) was determined in a randomly designated 1×1m subplot within each 5×5m plot at peak biomass. In the 1×1m subplot, cover was estimated visually to the nearest 1% for every species overhanging the subplot. Data on plant diversity were collected at all 84 sites.
Live vascular plant aboveground biomass (called plant biomass hereafter) was estimated destructively by clipping at ground level all aboveground biomass of plants rooted within two 1×0.1m strips (for a total of 0.2m2) adjacent to the 1×1m subplot where plant species diversity was determined. All biomass was dried at 60°C to constant mass before weighing to the nearest 0.01g. Data on plant biomass were collected at 74 of the 84 sites.
Soil samples were collected in the 5 × 5 m plots by taking three soil cores (2.5 cm diameter) at a depth of 0–10 cm. The three cores were pooled to make one sample per plot, air-dried and analyzed for total organic carbon (called soil organic carbon or soil carbon hereafter) and total nitrogen using an elemental analyzer (Costech ECS 4010 CHNSO Analyzer). Soil texture, expressed as the percentage sand, percentage silt, and percentage clay, was measured on 100 g dry soil using the Bouyoucos method. All soil samples were analyzed in the same laboratory (A&L Analytical Laboratory). Data on soil organic carbon (SOC) and soil nitrogen were collected at all 84 sites, data on soil texture were collected at 62 sites.
Plant species diversity (called plant diversity hereafter) was determined in a randomly designated 1×1m subplot within each 5×5m plot at peak biomass. In the 1×1m subplot, cover was estimated visually to the nearest 1% for every species overhanging the subplot. Data on plant diversity were collected at all 84 sites.
Live vascular plant aboveground biomass (called plant biomass hereafter) was estimated destructively by clipping at ground level all aboveground biomass of plants rooted within two 1×0.1m strips (for a total of 0.2m2) adjacent to the 1×1m subplot where plant species diversity was determined. All biomass was dried at 60°C to constant mass before weighing to the nearest 0.01g. Data on plant biomass were collected at 74 of the 84 sites.
Soil samples were collected in the 5 × 5 m plots by taking three soil cores (2.5 cm diameter) at a depth of 0–10 cm. The three cores were pooled to make one sample per plot, air-dried and analyzed for total organic carbon (called soil organic carbon or soil carbon hereafter) and total nitrogen using an elemental analyzer (Costech ECS 4010 CHNSO Analyzer). Soil texture, expressed as the percentage sand, percentage silt, and percentage clay, was measured on 100 g dry soil using the Bouyoucos method. All soil samples were analyzed in the same laboratory (A&L Analytical Laboratory). Data on soil organic carbon (SOC) and soil nitrogen were collected at all 84 sites, data on soil texture were collected at 62 sites.

Timing and spatial scale
The data were collected between 2nd January 2007 and 20th December 2020.
The 84 grassland sites explored in this study are natural and semi-natural grasslands located on six continents.
At each of the 84 grassland sites, on average 30 plots (measuring 5 × 5 m) were analyzed (see Table S1).

Data exclusions
No data were excluded.

Reproducibility
The study is based on observational data (i.e., no experimental manipulation). All measurements at all plots were done according to the same protocol (Borer et al., 2014).

Randomization
There was no experimental treatment involved in this study.

Blinding
Sample identity and study design were not disclosed to technical personal involved in the analysis of plant biomass and soil samples.

Did the study involve field work? ☒ Yes ☐ No

Field work, collection and transport

Field conditions
Across the 84 grassland sites, MAT ranges from -7.57 °C to 24.45 °C, MAP ranges from 192 mm to 2566 mm, and the aridity index ranges from 0.107 to 2.709. The 84 sites represent 19 grassland types (Table S1). All 84 sites are part of the Nutrient Network Global Research Cooperative (1) (NutNet, https://nutnet. org; Borer et al., 2014).

Location
The 84 grassland sites explored in this study are natural and semi-natural grasslands located on six continents, covering a wide range of climatic conditions (Table S1, Fig. S1). All 84 sites are part of the Nutrient Network Global Research Cooperative (1) (NutNet, https://nutnet. org; Borer et al., 2014).

Access & import/export
Samples were collected from grassland sites located on research stations or land owned by universities and research institutes. Biomass was determined locally by local staff.

Disturbance
Disturbance in the field was minimized (by avoiding to step on the plots as much as possible).

Reporting for specific materials, systems and methods

We require information from authors about some types of materials, experimental systems and methods used in many studies. Here, indicate whether each material, system or method listed is relevant to your study. If you are not sure if a list item applies to your research, read the appropriate section before selecting a response.

Materials & experimental systems

n/a	Involved in the study
☒	☐ Antibodies
☒	☐ Eukaryotic cell lines
☒	☐ Palaeontology and archaeology
☒	☐ Animals and other organisms
☒	☐ Clinical data
☒	☐ Dual use research of concern
☒	☐ Plants

Methods

n/a	Involved in the study
☒	☐ ChIP-seq
☒	☐ Flow cytometry
☒	☐ MRI-based neuroimaging
